# Self-induced *Elizabethkingia meningoseptica *endophthalmitis: a case report

**DOI:** 10.1186/1752-1947-5-303

**Published:** 2011-07-11

**Authors:** Paul P Connell, Sanj Wickremasinghe, Uma Devi, Mary Jo Waters, Penelope J Allen

**Affiliations:** 1 Centre for Eye Research Australia, The Royal Victorian Eye and Ear Hospital, University of Melbourne, 32 Gisborne Street, East Melbourne, Victoria 3002, Australia; 2 Vitreo-Retinal Unit, The Royal Victorian Eye and Ear Hospital, 32 Gisborne Street, East Melbourne, Victoria 3002, Australia; 3 Department of Microbiology, St Vincent's University Hospital, Melbourne, Victoria 3065, Australia

## Abstract

**Introduction:**

Endophthalmitis is a sight-threatening condition defined as any inflammation of the internal ocular spaces. It is classified as either endogenous or exogenous depending on the route of infection. Exogenous endophthalmitis results from direct inoculation as a complication of intra-ocular surgery, penetrating ocular trauma, intra-ocular foreign bodies, corneal ulceration and following a breach of ocular barriers from a periocular infection. We report a rare case of exogenous endophthalmitis with both unusual etiology and microbiology.

**Case presentation:**

A 41-year-old Caucasian man with a history of depressive illness presented to our eye department with painful acute visual loss on a background history of chronic uveitis. Ocular examination revealed a dense fibrinous panuveitis with a suspicion of a focal lesion in the posterior segment. Microbiological sampling from his anterior chamber and posterior segment revealed a culture of *Elizabethkingia meningoseptica*. On closer questioning, he volunteered the occurrence of multiple episodes of deliberate needle ocular penetration. Following vitrectomy for associated retinal detachment, a final Snellen visual acuity of 6/60 was obtained.

**Conclusions:**

*Elizabethkingia meningoseptica *endophthalmitis is a rare condition, and visual results to date are poor.

## Introduction

Endophthalmitis is a sight-threatening condition defined as any inflammation of the internal ocular spaces [1]. It is classified as either endogenous or exogenous depending on the route of infection. Exogenous endophthalmitis results from direct inoculation as a complication of intra-ocular surgery, penetrating ocular trauma, intra-ocular foreign bodies, corneal ulceration or following a breach of ocular barriers from a periocular infection [2]. Although endophthalmitis often presents with characteristic symptoms and signs, the differentiation between a pan-inflammatory condition and endogenous endophthalmitis can often be difficult, particularly in the absence of an overt exogenous cause.

We report a rare case of exogenous endophthalmitis with both unusual etiology and microbiology.

## Case presentation

**Figure 1:**
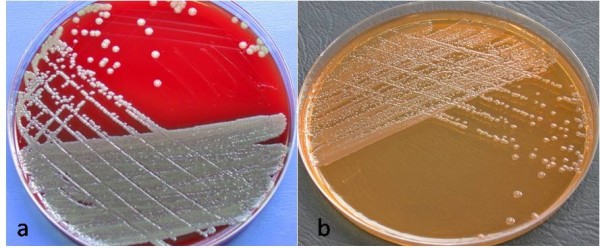
***Elizabethkingia meningoseptica *on (a) sheep blood agar and (b) MacConkey agar**.

**Figure 2:**
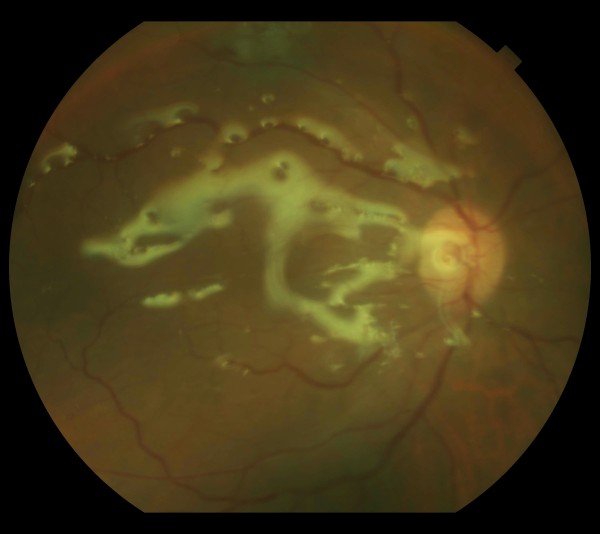
**Fundal appearance following vitrectomy and silicone oil tamponade**.

A 41-year-old Caucasian man presented to our eye department following ophthalmic referral with a three-day history of increasing pain and decreased visual acuity. His ocular history included recurrent seronegative uveitis, diagnosed two years previously and treated episodically with topical steroid therapy. He had been reviewed three days earlier by his ophthalmologist and commenced on 75 mg of prednisolone daily (with additional topical therapy) for a flare-up of his disease. He denied any history of trauma. His medical history was remarkable due to a 20-year history of depressive illness associated with episodes of deliberate self-harm. Ocular examination at presentation revealed a Snellen visual acuity of 2/60, conjunctival injection, +4 cells and flare in the anterior chamber associated with 30° of posterior synechiae formation and secondary cataract. No scleral perforation, penetration site or corectopia was observed. Fundal examination revealed a dense vitritis with an indistinct yellow lesion in the inferior retina. A differential diagnosis of endogenous, post-traumatic (given his medical history) or infective endophthalmitis was contemplated. Laboratory investigations revealed a normal full blood count and viral titer results were negative. Blood culture results were also negative. At one hour after admission, a 25 g aspirated vitreous biopsy was performed (for Gram staining, culture and sensitivities and viral PCR) with intra-vitreal ceftazidime and vancomycin administered. Polymorphs and Gram-negative rods were seen in a Gram stain from the vitreous tap. After 48 hours, a heavy growth of a Gram-negative, oxidase-positive rod was detected on sheep blood agar and MacConkey agar (Figure 1). The isolate was subsequently identified as *Elizabethkingia meningoseptica *using a VITEK 2 system. Due to the unusual nature of the isolated microbe and upon closer and repeated questioning, our patient volunteered the occurrence of multiple episodes of deliberate ocular penetration with an unsterile sewing needle on more than 20 occasions over the preceding nine months. At three days after admission, on suspicion of an inferior retinal detachment, he underwent a 23 g pars plana vitrectomy with repeat intra-vitreal antibiotic therapy. A macular intra-retinal abscess was detected at surgery with no retinal detachment. Upon close follow-up, his intra-ocular inflammation improved upon topical steroid and antibiotic therapy. With the isolation of an organism inherently resistant to many classes of antimicrobials he was commenced on a six-week course of oral rifampicin and ciprofloxacin therapy, based on sensitivity test results and a review of the literature. At two weeks after initial surgery he presented again with a rhegmatogenous retinal detachment necessitating pars plana vitrectomy, lensectomy, endolaser and silicone oil (Figure 2). At four weeks after surgery the retina remained flat with trace inflammatory change with a Snellen visual acuity of 6/60. A psychiatric consult was also arranged at this time.

## Discussion

Penetrating eye injuries predominantly occur in younger men and are a common cause of monocular visual loss. Self-inflicted eye injuries as a cause of visual loss are an unusual, but important, form of self-mutilation [3,4]. They have been associated with a variety of psychological disorders, including paranoid schizophrenia [5-7], drug-induced psychosis, obsessive-compulsive disorder [7], depression, mental retardation [6] and ritualistic behavior [8-10] and organic illnesses [8], including neurosyphilis [11], Lesch-Nynan syndrome [12], and following organic brain injury [13]. Needle perforation is an uncommon form of self-mutilation. It has been described in both adults and children, but occurs most commonly in younger adults with acute and chronic psychoses. Figures pertaining to self-inflicted needle perforations are unavailable due to the sporadic nature of the reports. Soylu *et al*. reported a non-self-inflicted perforation frequency of 8.3% in one pediatric population, with no figures pertaining to the adult population [12].

Ocular injuries pertaining to deliberate self-harm include self-enucleation, orbital trauma, injury to the ocular surface and anterior segment, and posterior segment injury. Few reports pertain to deliberate self-harm with a periocular or ocular injection, particularly in middle-aged men [14,15]. Ang *et al*. reported a case of bilateral penetrating ocular trauma secondary to a self-inflicted injury with a hypodermic needle in a 12-year-old [5].

The unusual nature of the microbe isolated in this case prompted further questioning of our patient to ascertain the pathogenesis and etiology of the presenting endophthalmitis, especially given the unfortunate medical history. *Elizabethkingia meningoseptica *(previously called *Flavobacterium meningosepticum *and more recently *Chryseobacterium meningosepticum*) is a Gram-negative, non-motile, oxidase-positive, catalase-positive rod that produces proteases and gelatinases that contribute to virulence. *Elizabethkingia menigoseptica *has reduced susceptibility to a broad range of antimicrobials, including beta lactams, aminoglycosides and chloramphenicol. *In vitro *studies examining isolates from neonatal infections suggested a variety of drug combinations may be useful in treating *Elizabethkingia meningoseptica *infections, ciprofloxacin and rifampicin were among the drugs considered [14]. *Elizabethkingia meningoseptica *is normally found associated with water and soil, food products and, at times, in the hospital environment. It is an opportunistic pathogen being most often associated with meningitis and septicemia in a pediatric population [15]. The organism is ubiquitous and environmental contamination can occur readily, as occurred here in relation to the contaminated needle. In the 1960s and 1970s cases of endocarditis and pneumonia attributed to *Elizabethkingia meningoseptica *were reported in the literature. A limited number of reports have documented bacteremia in adults, with the largest documented case series to date in a Taiwanese population. This study of 32 patients, demonstrated variable susceptibilities to antibiotic therapy with a mortality rate of 28%. None had presented with an intra-ocular infection. We previously identified one case of post-traumatic *Elizabethkingia meningoseptica *endophthalmitis following a penetrating eye injury following a road traffic accident [14]. To the best of our knowledge, the present case is the first documented case demonstrating endophthalmitis following deliberate self-harm in an adult with a poor visual outcome.

## Conclusions

Self-inflicted ocular trauma as a cause for visual loss is unusual. The nature of the offending pathogen may point to a potential etiological cause, but does not replace a thorough medical and social history.

## Consent

Written informed consent was obtained from the patient for publication of this case report and any accompanying images. A copy of the written consent is available for review by the journal's Editor-in-Chief.

## Competing interests

The authors declare that they have no competing interests.

## Authors' contributions

PC examined and interpreted patient data at presentation and performed the surgical operations. SW also examined our patient with particular reference to the associated history of deliberate self-harm. UD provided physician support. MJW provided clinical and microbiological support and isolated the offending pathogen. PA examined and assisted at surgery as lead consultant. All authors read and approved the final manuscript.
